# Supplementary figures and images for: A Cellular Senescence-Related Signature Predicts Cervical Cancer Patient Outcome and Immunotherapy Sensitivity
Source: Reprod Sci. 2023 Aug 14;30(12):3661–76. doi: 10.1007/s43032-023-01305-w (PMC10691978; doi:10.1007/s43032-023-01305-w)

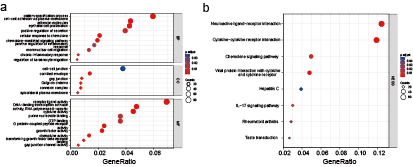

Supplement: Supplementary file 1 — Fig. S1. GO (a) and KEGG (b) enrichment analysis of DEGs between normal and tumor samples. (PNG 20 kb) [file 43032_2023_1305_Fig10_ESM.png]

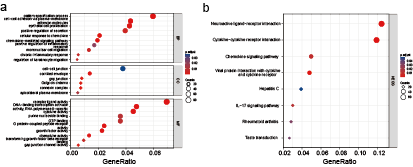

Supplement: Supplementary file 2 — High resolution image (TIF 123 kb) [file 43032_2023_1305_MOESM1_ESM.tif]

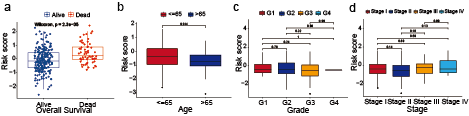

Supplement: Supplementary file 3 — Fig. S2. Correlations between the risk model and diverse clinical parameters (a) Survival status, (b) Age, (c) Grade, (d) Stage. (PNG 18 kb) [file 43032_2023_1305_Fig11_ESM.png]

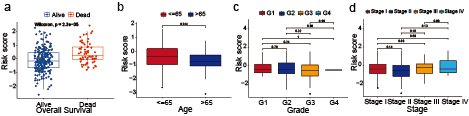

Supplement: Supplementary file 4 — High resolution image (TIF 80 kb) [file 43032_2023_1305_MOESM2_ESM.tif]

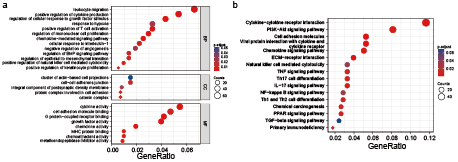

Supplement: Supplementary file 5 — Fig. S3. GO (a) and KEGG (b) enrichment analysis of DEGs between high-risk and low-risk cervical cancer patients. (PNG 27 kb) [file 43032_2023_1305_Fig12_ESM.png]

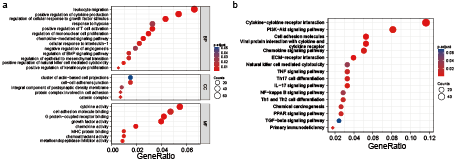

Supplement: Supplementary file 6 — High resolution image (TIF 144 kb) [file 43032_2023_1305_MOESM3_ESM.tif]
